# New treatments for the mucopolysaccharidoses: from pathophysiology to therapy

**DOI:** 10.1186/s13052-018-0564-z

**Published:** 2018-11-16

**Authors:** Simona Fecarotta, Serena Gasperini, Giancarlo Parenti

**Affiliations:** 1 Department of Translational Medical Sciences, Federico II University, Naples, Italy; 2 Metabolic Rare Disease Unit, Pediatric Department, Fondazione MBBM, University of Milano Bicocca, Monza, Italy; 3 Telethon Institute of Genetics and Medicine, Pozzuoli, Italy

**Keywords:** Mucopolysaccharidoses, Enzyme replacement therapy, Gene therapy, Blood-brain barrier, Autophagy

## Abstract

Enzyme replacement therapy is currently considered the standard of care for the treatment of mucopolysaccharidoses (MPS) type I, II, VI, and IV. This approach has shown substantial efficacy mainly on somatic symptoms of the patients, but no benefit was found for other clinical manifestations, such as neurological involvement. New strategies are currently being tested to address these limitations, in particular to obtain sufficient therapeutic levels in the brain. Intrathecal delivery of recombinant enzymes or chimeric enzymes represent promising approaches in this respect. Further innovation will likely be introduced by the recent advancements in the knowledge of lysosomal biology and function. It is now clear that the clinical manifestations of MPS are not only the direct effects of storage, but also derive from a cascade of secondary events that lead to dysfunction of several cellular processes and pathways. Some of these pathways may represent novel therapeutic targets and allow for development of novel or adjunctive therapies for these disorders.

## Background

Over the past two decades, extraordinary advancements have been achieved in the treatment of lysosomal storage diseases, including the mucopolysaccharidoses (MPS). Different approaches have been designed to treat these disorders and, for several of them, enzyme replacement therapy (ERT) is currently considered the standard of care; however, clinical research is rapidly moving towards other advanced approaches, such as gene therapy.

Despite considerable success in treating some lysosomal storage diseases, however, current therapies have been unable to cure all the pathological and clinical manifestations of the diseases, leaving several unmet medical needs. Current research is now trying to address these needs, and to improve the efficacy of existing therapies. In this respect it is of key importance to understand in detail the cascade of events and the cellular processes that ultimately lead to tissue pathology and clinical manifestations of the MPS.

The research and the growing expansion of our knowledge in the field of lysosomal storage diseases and MPS represent excellent models of the renewed interest for the biology and the mechanisms of diseases in many other fields of pediatrics. For other pediatric disorders, such as muscular dystrophies, rheumatological diseases, and genetic immunodeficiencies, current research is now focused on the search for “biological” drugs, precisely directed towards specific cellular pathways.

The scope of this article is to review current and new treatment strategies in MPS, with a particular focus on the limitations and challenges imposed by existing therapies with unmet needs. The knowledge of the secondary pathways that are implicated in the pathophysiology of MPS is the basis for the development of new treatment strategies that are then discussed.

## The changed vision of lysosomal biology and its implications for the pathophysiology of MPS

Since their discovery by Christian de Duve [1], lysosomes have been viewed as organelles exclusively specialized in degradative functions. This vision of lysosome biology, however, has dramatically changed in recent years. A number of studies have provided compelling evidence that lysosomes are not only organelles specialized for the catabolism of complex macromolecules, but they are actively involved in many critical cellular processes, such as signaling pathways, secretion, vesicle and plasma membrane trafficking, regulation of metabolism, growth, adaptive immunity, and others [2].

The new vision of lysosomal function has translated into a better understanding of the pathophysiology of lysosomal storage diseases, including the MPS that are due to the deficiency of lysosomal hydrolases involved in the breakdown of glycosaminoglycans (GAGs).

**Fig. 1:**
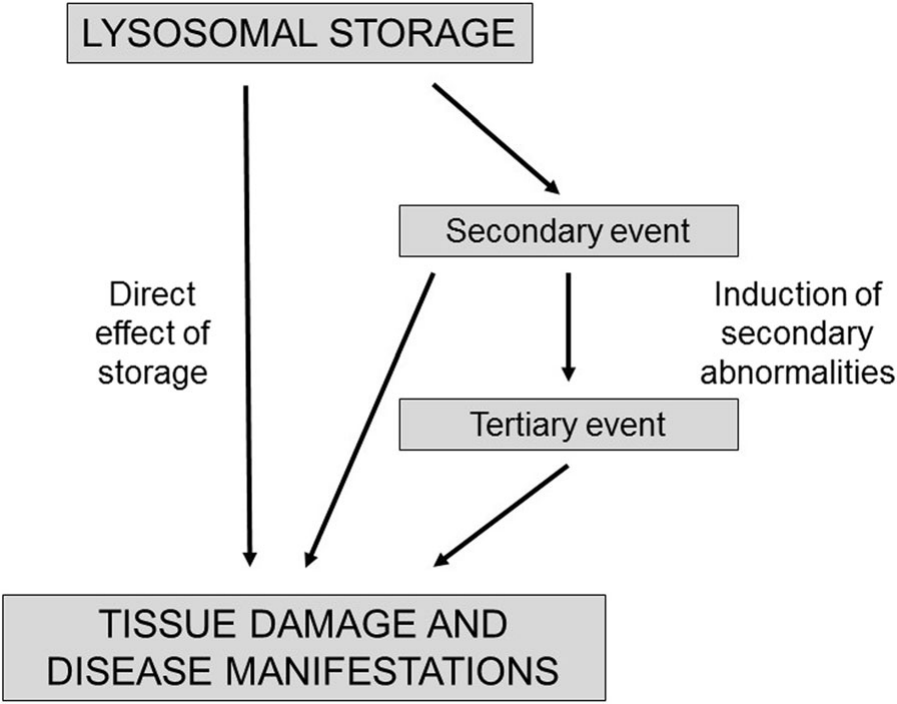
The pathology and the clinical manifestations of mucopolysaccharidoses are only in part the direct consequences of the storage of substrates. A new vision of lysosomal disease pathophysiology suggests that secondary and tertiary events, such as activation of cellular pathways, significantly contribute to the occurrence of tissue damage and clinical manifestations

Historically, the pathology and the clinical manifestations of lysosomal disorders have been considered as direct consequences of the storage of inert substrates in tissues. Given the newly recognized roles of lysosomes in cellular homeostasis and metabolism, however, it is reasonable to speculate that storage is rather the “instigator” of a number of secondary events [3] that are triggered by the accumulation of undegraded substrates and that significantly contribute to complex pathogenetic cascades (Fig. 1).

**Fig. 2:**
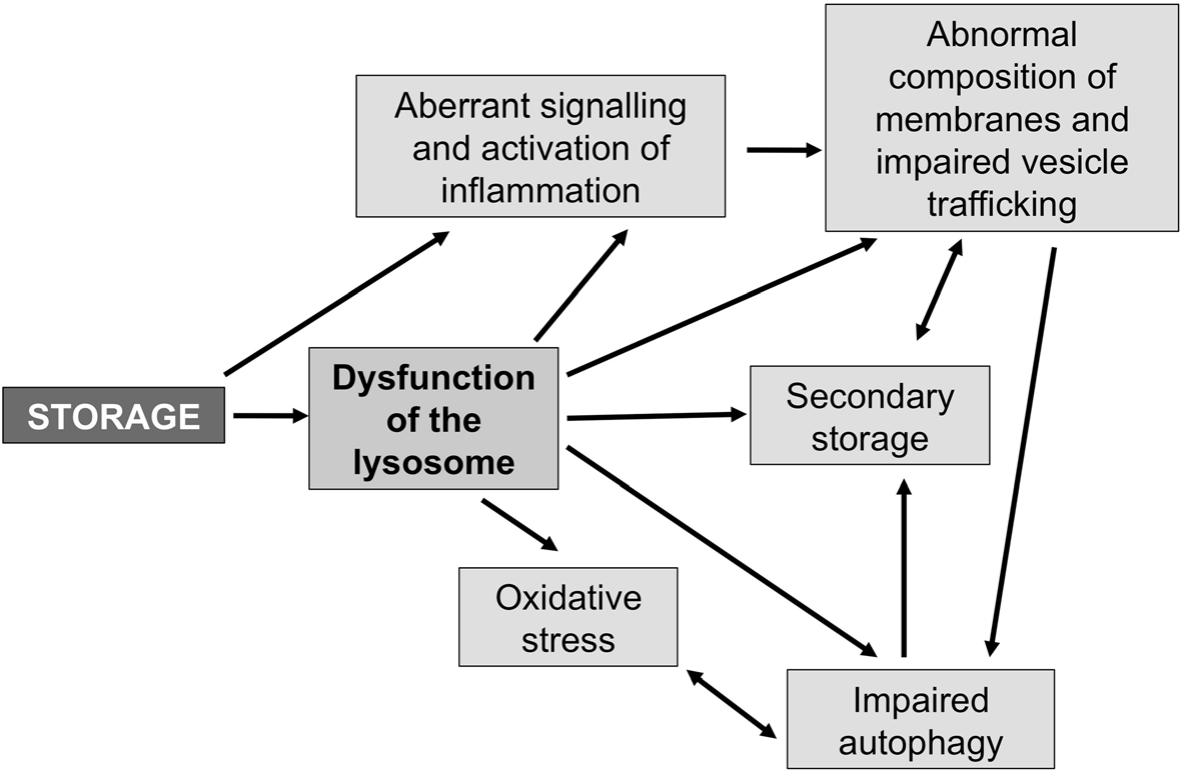
Several studies suggest that in mucopolysaccharide storage causes dysfunction of lysosomes and secondary events, such as aberrant activation of signaling pathways, impairment of autophagy, abnormal vesicle and plasma membrane trafficking, etc

This hypothesis has been largely confirmed by recent studies. Indeed, a broad range of events are now emerging as major players in the pathogenesis of MPS. These include: storage of secondary substrates unrelated to the defective enzyme and abnormal composition of membranes; aberrant fusion and intracellular trafficking of vesicles, membranes, and membrane proteins; impairment of autophagy; alteration of signaling pathways; oxidative stress; abnormalities of calcium homeostasis; and several others [4–7]. The dysfunction of each of these pathways impacts and influences the others with an intricate interplay, thus contributing to the complexity of the pathological, functional, and phenotypic manifestations of the MPS and of other lysosomal storage diseases (Fig. 2).

## Why it is important to know the pathophysiology of MPS?

The improved understanding of the pathophysiology of lysosomal storage diseases, and in particular of the MPS, has evident and critical implications for the treatment of these disorders. For decades, the primary approach to their treatment has been based on restoring the equilibrium of the so-called “storage equation”, that is the balance between the amount of substrate that is delivered to lysosomes for degradation and the amount of enzymes that are involved in its breakdown. To this end, different therapeutic approaches were designed, either to reduce the flux of substrates to lysosomes (substrate reduction therapy), or to provide normal and functional enzymes from external sources (ERT, hematopoietic stem cell transplantation, gene therapy). These approaches have proved to be successful in treating some diseases or some manifestations of individual disorders.

However, after many years of experience and based on the data collected by international registries, it has become clear that these approaches have important limitations, particularly in terms of biodistribution of therapeutic agents, limited efficacy in specific tissues, fluctuating levels of activity (for ERT), impact on the quality of life of patients and their carers, and cost, and that several challenges remain to achieve a complete cure of patients affected by MPS. For example, important target tissues in patients with MPS (such as the brain, bone, heart, and eye) appear to be refractory to ERT due to the inability of the recombinant enzymes to reach therapeutic levels in these tissues. Recombinant enzyme preparations are highly expensive (up to several hundred thousand euros for the treatment of a single patient per year) [8]. Finally, weekly parenteral administrations of recombinant enzymes has a heavy impact on the quality of life of patients and their families and often require intravenous devices with consequent risks of infection.

The characterization of the cellular processes that are involved in the pathophysiology of lysosomal diseases is now providing clues to address the limitations of the existing therapies, and to identify additional therapeutic targets. In principle, some of the pathways that are perturbed in the MPS may be pharmacologically manipulated and may represent novel therapeutic targets, potentially translating into adjunctive and effective tools to treat patients.

## Improving the targeting of corrective enzymes to the central nervous system

ERT has been effective in decreasing GAG storage in viscera and the heart, reducing or normalizing GAG urinary excretion, and improving joint mobility, endurance, and respiratory function. On the other hand, it has become clear that ERT has a limited impact on other tissues and organs, such as cardiac valves, bone, and cornea. Of even greater importance for the outcome of patients is the inability of the recombinant enzymes used for ERT to cross the blood–brain barrier and to cure or alleviate cognitive decline and spinal cord compression, especially in MPS I, II, III, VI, and VII in which neurological involvement is often a critical and debilitating manifestation.

For these reasons, the search for new and more effective therapeutic strategies that may address the limitations of ERT has become a major field of research in the last years and has attracted commercial interest from the pharmaceutical industry [9].

Predicted and feasible routes of administration are based on direct delivery of the therapeutic enzyme into the brain. This can be achieved either by intraparenchymal or intrathecal/intraventricular injection. The lumbar approach permits getting closer to the brain by exploiting the cerebrospinal fluid (CSF) flow in a less invasive way. The concept of intrathecal ERT is based on the rationale that tiny amounts of enzyme could cross the ependymal layer and create a large concentration gradient of enzyme in the brain tissue by a very efficient uptake process [10].

Thus far, many invasive strategies have been exploited. The availability of animal models (especially mice and dogs) that recapitulate MPS human pathophysiology was pivotal in this respect [11] and allowed for translation into a few clinical trials. The results of these studies, however, were variable. Initially, chemical or physical agents were used to cross the blood–brain barrier (as mannitol), but they proved unable to lead the enzyme into the ependymal layer and diffuse through brain tissue [12].

Intrathecal therapy was then selected as a strategy for delivering the recombinant enzymes across the ependyma and to by-pass the obstacle of the blood–brain barrier [13]. In animal models this approach showed promising results and gave a substantial impulse for subsequent clinical trials in MPS I, II, IIIA, and VI [14–16]. In particular, a study in MPS I dogs showed that the enzyme can distribute throughout the neuraxis, achieve extremely high enzyme levels in the spinal cord and meninges, reduce GAG accumulation, and decrease storage in the spinal anterior horn cells. In dogs, clinical improvement in terms of gait due to cord compression were observed [17].

ERT delivery into the cisterna magna was tested in New Zealand Huntaway dogs, an animal model of MPS IIIA. This strategy led to widespread diffusion of the enzyme in areas of the brain and spinal cord, with evidence of penetration into the deeper cortex and brain structures that are not in direct contact with the CSF, such as white matter, basal ganglia, hippocampus, and thalamus [18]. A preclinical study based on intrathecal ERT was also performed in MPS IIIA mice; the ventricular route revealed great efficacy, even though it was more invasive [19].

Despite the encouraging preclinical data in animal models, little and erratic progress has been made when the clinical trials started in humans [20, 21]. Intrathecal infusion of the enzyme was tested in human therapy for MPS I, II, IIIA, and VI [21–23] in phase I/II clinical trials. The main problems in humans are the short half-life of the enzyme, its low efficacy, a substantial risk of infections, and cellular and humoral responses to enzymes. Furthermore, these approaches require invasive procedures such as frequent sedations (which in MPS patients are associated with major risks) and surgical implantation of intrathecal drug delivery devices (IDDD). Many devices were studied to reduce the complications of an invasive technique, such as the SOPH-A-PORT mini S device.

Monthly intrathecal infusions via lumbar puncture were first described in 2008 by Munoz-Rojas et al. [14] in a single patient affected by MPS I Hurler-Scheie. The patient showed improvements in terms of stability and gait control and respiratory parameters, and no major adverse events occurred. Another study in a 23-year-old male MPS I Hurler-Scheie patient showed better neurocognitive performance (in terms of memory, attention, and learning functions) after intrathecal therapy [24]. An immune response to intrathecal ERT was observed in MPS I patients [25]. Another study in MPS II patients combined intravenous and intrathecal revised formulation of the enzyme idursulfase suitable for intrathecal delivery [23]. The intrathecal administration via IDDD in patients with Sanfilippo Syndrome type A (MPS IIIA) initially showed clinical safety, albeit in the presence of antibody formation and decline of CSF heparan sulfate levels [26], but after a phase IIb trial this therapy was judged ineffective, particularly in terms of cognitive profile.

The intrathecal approach has also been used to prevent cord compression due to dural and leptomeningeal thickening for GAG accumulation in a patient affected by MPS I [14].

Other strategies to directly penetrate the brain are under evaluation, including intravenous ERT with chimeric fusion proteins as a sort of “Trojan horse”. With this approach, the recombinant enzymes are engineered as a chimeric protein by the fusion of the enzymatic sequencing with specific peptides. Peptides are fragments of a protein that are recognized by specific receptors and can transport the therapeutic enzyme across nonphysiological and different pathways with resulting better access to the central nervous system. Preclinical studies with animal models were performed using antibodies to antitransferrin receptor or human insulin-like growth factor in MPS I, MPS IIIA [27–29], and MPS II [30]. A more recent approach uses conjugation instead of fusion [31]. This approach is also used to improve the efficacy of gene therapy [32].

Subcutaneous implantation of microencapsulated cells overexpressing α-l-iduronidase is another promising cell therapy method in MPS I that has been tested in a murine model of the disease [33].

Recently, intravenous ERT has also become available for patients affected by MPS VII or Sly Syndrome. A phase III trial showed good results in the first patient treated, with dramatic GAG and hepatosplenomegaly reduction or normalization, and improvements in growth and pulmonary function with a reduction in ventilatory parameters and oxygen requirement [34].

## Storage and substrate reduction

While ERT aims to decrease the primary storage of GAGs by increasing their degradation through the exogenous administration of recombinant enzymes, a different therapeutic approach (commonly referred to as “substrate reduction therapy”) is aimed at reducing the excess of substrate by inhibiting its synthesis. This approach has potential advantages compared with enzyme replacement. Inhibitors of substrate synthesis are small molecules that are able to cross the blood–brain barrier and may have the potential to treat central nervous system involvement in the neurological forms of MPS.

GAG synthesis is a complex process that requires a large number of sequential steps. The first steps lead to the covalent addition of a tetrasaccharide chain onto a serine residue of the core protein. Each of these steps is catalyzed by specific glycosyl- and sulfo-transferases. The oligosaccharide chain is then elongated by other enzymes, and its composition diverges towards the formation of specific GAGs (heparan sulfate, dermatan sulfate, chondroitin sulfate, etc.).

The knowledge of the enzymes involved in GAG synthesis and of the mechanisms regulating this process has provided clues to the development of substrate reduction therapy for MPS. It has been shown that this process is regulated by the epidermal growth factor (EGF) receptor pathway since the EGF-mediated signal transduction regulates the expression of genes encoding the individual enzymes involved in GAG production. Genistein, a soy-derived isoflavon with structural similarity to 17β-estradiol, inhibits the tyrosine kinase activation of the EGF receptor, thus inhibiting the signal that leads to the transcription of the enzymes responsible for the GAG synthesis [35–38]. Genistein was thus the first molecule that was identified as a potential therapeutic target and proposed as a possible drug for substrate reduction therapy in patients with MPS and neurological manifestations. After preclinical evaluation, this approach, known as gene expression-targeted isoflavone therapy (GET IT), has been translated into clinical trials in MPS III patients.

Pilot studies with orally administered genistein at the dose of 5 mg/kg/day [39, 40] showed reduction in GAG excretion in combination with improvements in behavior and a reduced rate of neurological deterioration in MPS III treated patients. The authors of this study concluded that results were promising and speculated that higher doses of genistein may further enhance the efficacy of treatment.

However, subsequent experiences were less encouraging. A randomized controlled cross-over trial with oral genistein at a dose of 10 mg/kg/day in 30 patients with MPS III only showed a temporary reduction in GAG excretion in the absence of any clinical effects in treated patients [41].

Further studies showed that higher doses of genistein (up to 150 mg/kg/day) are safe, but clinical efficacy remains uncertain with a need for further evaluation in the long term and in selected patient series [42]. Although treatment with genistein has been mainly proposed for the treatment of neurological manifestations in patients with MPS in the absence of therapeutic alternatives, some authors have administered genistein in seven MPS II patients who lacked ERT as an option, showing efficacy in connective tissue elasticity and, particularly, in improving the range of joint motion [43].

Although genistein has so far failed to show significant improvements in neurological outcome, substrate reduction therapy remains an attractive approach to treat MPS, and it is possible that the identification of novel drugs (such as inhibitors of individual steps of GAG synthesis) may provide better results.

In addition to primary storage, in several MPS there is secondary storage of substrates that are not clearly related to the primary lysosomal defect. Secondarily stored compounds are highly heterogeneous and include lipids, glycosphingolipids and phospholipids, and cholesterol [44, 45]. Secondary storage was for a long time considered a nonspecific and insignificant pathologic feature of the MPS. However, recent studies indicate that secondarily stored compounds may also play a role in the disease pathogenesis. Secondary accumulation of particular classes of compounds may explain some features of the disease pathology and some clinical manifestations, such as activation of inflammation. In principle, reducing the levels of these compounds may be an adjunctive therapeutic strategy in MPS and may result in beneficial effects in patients.

According to this assumption, some studies have identified secondary storage of gangliosides as a therapeutic target in MPS patients with neurological involvement. Miglustat (*N*-butyl-deoxynojirimycin, NB-DNJ) is an imino sugar that inhibits the synthesis of glucosylceramide, which is the precursor for the synthesis of Gm1 and Gm2 gangliosides. This drug is a small molecule that is able to cross the blood–brain barrier and is currently approved for the treatment of two other lysosomal storage disorders, Gaucher disease and Niemann-Pick disease type C. Preclinical studies in animal models confirmed a possible efficacy of this therapeutic approach, showing an improvement in the behavior of treated animals and the reduction of Gm2 ganglioside levels and of neuroinflammation [46].

The efficacy of miglustat in MPS III patients has been evaluated in a double-blind, randomized controlled clinical trial using appropriate scales to assess the clinical endpoints as behavioral disorders, sleep disorders, and hyperactivity [47]. The selected dose of miglustat was the same as used in patients with Niemann-Pick type C disease, with the aim to reach therapeutic concentrations of the drug in the CSF. Despite the attractive premise and the preclinical studies, however, the results of the trial were disappointing, and there was no increase in the percentage of clinical success in the treated group compared with the placebo group of patients. Moreover, the levels of the gangliosides in the CSF were not significantly decreased in the two groups. The reduction of secondary substrates, such as Gm2 gangliosides, with miglustat remains to be evaluated in the future in selected patient series.

## Manipulation of proinflammatory pathways

An important, and somehow unexpected, effect of GAG accumulation in the MPS is the nonphysiologic activation of signal transduction pathways, particularly of inflammation. Several studies performed both in animal models and humans suggest that this mechanism is implicated in the pathophysiology of some of the most debilitating manifestations of these disorders, such as central nervous system involvement and skeletal abnormalities, and that may represent an additional target for therapy.

Incompletely degraded or structurally different GAGs may mimic lipopolysaccharide (LPS), an endotoxin of gram-negative bacteria which is known to bind and activate the Toll-like receptor (TLR)4. Activation of TLR4, in turn, promotes secretion of proinflammatory cytokines, overexpression of the LPS-binding protein, and of MyD88 [48], and activation of tumor necrosis factor (TNF)-alpha. Elevated levels of TNF-alpha and other inflammatory markers in this pathway have been reported in animal models of MPS and in patients with MPS I, II, and III [49]. Elevated levels of osteopontin, a regulator of inflammation, bone mineralization, and activation of macrophages, were found in serum obtained from children with MPS [50].

In the central nervous system, proinflammatory conditions are associated with activation of microglia, secretion of inflammatory cytokines and chemokines including TNF-alpha, interleukin (IL)-1β, IL-6, and macrophage inflammatory protein-1-alpha (CCL3) [51], leading to chronic brain inflammation [52]. In a mouse model of MPS VII, analysis of the gene expression profile revealed upregulation of the immune system, activation of inflammation, and downregulation of major oligodendrocyte genes, with specific patterns in different regions of the brain [53].

The characterization of these abnormalities has evident and immediate therapeutic implications. In particular, the TLR4-TNF-alpha pathway has been recognized as a potential therapeutic target.

Simonaro et al. [48] showed that activation of TLR4 signaling resulted in altered STAT1 and STAT3 expression, with a pattern similar to that observed in rheumatoid arthritis, leading to increased TNF-alpha levels in MPS tissues. Based on these data, it was speculated that treatment with anti-TNF-alpha drugs might improve bone and joint manifestations in MPS. Infliximab was tested in experimental animal models of MPS. MPS VI rats treated with infliximab intravenously showed a reduction in TNF-alpha serum levels and a parallel reduction in apoptotic cells in articular cartilage in comparison with untreated animals [48].

Infliximab also enhanced the efficacy of ERT in MPS VI rats [54]. Animals treated with ERT in combination with infliximab (at a dose of 3 mg/kg twice a week intravenously) showed significantly lower serum levels of TNF-alpha in comparison with untreated animals, and improved bone length and better motor performance on rotarod in comparison with the animals treated with ERT alone.

Based on these encouraging preclinical results, anti-TNF-alpha treatment has been translated to human therapy for the treatment of bone and joint manifestations in MPS, and clinical trials are currently recruiting MPS I, MPS II, and MPS VI patients to explore the safety and efficacy of subcutaneous adalimumab [55].

The anti-TNF-alpha approach may result in clinical benefit in MPS patients, mainly addressing the pathogenic mechanism of joint and bone disease that cannot be successfully treated with ERT. In the future, a combination treatment based on ERT in association with anti-TNF-alpha drugs could show therapeutic advantage in comparison with ERT alone, even if potential limits exist (for example, immunosuppression and adjunctive costs).

Pentosan polysulfate (PPS), a Food and Drug Adminsitration (FDA)-approved drug with anti-inflammatory and prochondrogenic properties, was shown to improve clinical manifestations of the disease in MPS VI rats, and to reduce urinary GAGs and proinflammatory cytokines (IL-8 and TNF-alpha) in tissues and in the CSF in MPS I dogs [48, 56, 57]. PPS also enhanced the effects of ERT in rats with mucopolysaccharidosis type VI [54] and was effective in improving the clinical outcome in MPS VI rats, even when treatment was used as monotherapy [56].

MPS VI rats orally treated with PPS showed decreased serum and tissue levels of TNF-alpha and other inflammatory biomarkers. PPS treatment was also useful for improving the hyaline cartilage of tracheas, resulting in improvement in tracheal anatomy. The treated animals also showed improvement in motor performance on the rotarod test and a reduction in the skull and dentition changes with a decrease in the typical coarse appearance [57].

A clinical study in humans [58] investigated the safety and the efficacy in terms of mobility and pain of PPS treatment in four MPS I patients in addition to ERT. PPS was well tolerated by all patients and resulted in a significant reduction in urinary GAG excretion and in an improvement in joint mobility and pain.

In the future, clinical studies could demonstrate the additional benefit of PPS treatment when used in combination with ERT, and future preclinical studies should also investigate if the effect of treatment on neural inflammation is similar to that of the anti-TNF-alpha drugs with reduced additional risks.

## The impairment of autophagy and other cellular pathways

Storage triggers abnormalities of several other processes that are involved in cellular homeostasis. The best characterized of these pathways is autophagy. Autophagy (from the ancient Greek for self-eating) is a cellular process that aims at delivering and catabolizing cellular components, such as proteins and organelles, that need to be recycled in the lysosomes. Once in the lysosome, proteins and organelles are degraded by the lysosomal hydrolytic enzymes. There is now substantial evidence indicating that the autophagic-lysosomal pathway is heavily affected in lysosomal storage diseases, including some MPS, and that these abnormalities are major determinants of the disease pathophysiology [59–61]. For example, the impairment of the autophagic flux has been recognized as an important pathogenic factor for neurodegeneration, one of the most debilitating manifestations of MPS [62]. In neurons, failure of autophagy results in secondary accumulation of toxic storage materials, including polyubiquitinated proteins, aggregate-prone proteins, and damaged mitochondria [63, 64]. Another consequence of the impairment of autophagy is the accumulation of dysfunctional mitochondria, likely one of the mechanisms underlying neurodegeneration in the MPS IIIC mouse model [65].

It has been speculated that manipulating the autophagic-lysosomal pathway (and correcting its perturbations in the lysosomal storage diseases) may represent a possible strategy for the development of new therapies for MPS. Indeed, preliminary studies in cells from MPS IIIA and multiple sulfatase deficiency (a disorder in which there is a simultaneous defect of all sulfatases, including those that are deficient in some MPS) seem to confirm this hypothesis. In these cells, overexpression of the gene encoding the transcription factor EB (*TFEB*), a master regulator of autophagy [66], resulted in near-complete clearance of GAGs [67].

Other studies, mostly performed in animal models, suggested a role of oxidative stress in the pathophysiology of some manifestations of the MPS [68, 69]. Increased oxidative stress was found in the mouse model of MPS IIIB, even in the early stages of the disease course. Oxidative imbalance was also found in animal models of MPS IVA [70] and MPS IIIA [71], and in patients affected by MPS I [72].

Other secondary abnormalities such as impaired calcium homeostasis, first described in lysosomal disorders such as Niemann-Pick disease type C, were observed in MPS. These abnormalities are particularly relevant as acidic calcium stores play a central role in the regulation of vesicle trafficking and fusion. Evidence of disruption of calcium and proton homeostasis was also shown in MPS I [73].

## Conclusion

The MPS remain an important medical challenge in terms of social burden for patients and their families and of economic costs. Thanks to the new technologies available, and to the effort of metabolic physicians and the pharmaceutical industry, some of the limitations of currently available therapies are being addressed. Even if the first results of strategies aimed at correcting the secondary abnormalities of cellular pathways are still very preliminary or disappointing, it is reasonable to expect that an improved characterization of the precise mechanisms implicated in the disease pathophysiology will help to develop new and complementary therapeutic strategies.

## Key points

- The understanding of physiopathology is necessary to map out new treatments
- New approaches have been used over the last years to treat patients affected by MPS thanks to significant progress in biomedical research, and new attractive challenges will follow
- Clinical trials are still under evaluation despite clinical success in animal models
- Significant unmet medical needs still exist
- This article examines some examples of these new therapeutic strategies

## Abbreviations

CSF: Cerebrospinal fluid
EGF: Epidermal growth factor
ERT: Enzyme replacement therapy
GAG: Glycosaminoglycan
IDDD: Intrathecal drug delivery devices
IL: Interleukin
LPS: Lipopolysaccharide
MPS: mucopolysaccharidosi(e)s
PPS: Pentosan polysulfate
TLR: Toll-like receptor
TNF: Tumor necrosis factor
